# Synthetic cranial MRI from 3D optical surface scans using deep learning for radiation therapy treatment planning

**DOI:** 10.1007/s13246-023-01229-4

**Published:** 2023-02-08

**Authors:** Michael Douglass, Peter Gorayski, Sandy Patel, Alexandre Santos

**Affiliations:** 1 Department of Radiation Oncology, Royal Adelaide Hospital, Adelaide, SA 5000 Australia; 2 Australian Bragg Centre for Proton Therapy and Research, SAHMRI, Adelaide, SA 5000 Australia; 3 School of Physical Sciences, University of Adelaide, Adelaide, SA 5005 Australia; 4 University of South Australia, Allied Health & Human Performance, Adelaide, SA 5000 Australia; 5 Department of Radiology, Royal Adelaide Hospital, Adelaide, SA 5000 Australia

**Keywords:** Deep learning, Photogrammetry, 3D scan, pix2pix, GAN, Synthetic, MRI, Radiation oncology, Treatment planning

## Abstract

**Background:**

Optical scanning technologies are increasingly being utilised to supplement treatment workflows in radiation oncology, such as surface-guided radiotherapy or 3D printing custom bolus. One limitation of optical scanning devices is the absence of internal anatomical information of the patient being scanned. As a result, conventional radiation therapy treatment planning using this imaging modality is not feasible. Deep learning is useful for automating various manual tasks in radiation oncology, most notably, organ segmentation and treatment planning. Deep learning models have also been used to transform MRI datasets into synthetic CT datasets, facilitating the development of MRI-only radiation therapy planning.

**Aims:**

To train a pix2pix generative adversarial network to transform 3D optical scan data into estimated MRI datasets for a given patient to provide additional anatomical data for a select few radiation therapy treatment sites. The proposed network may provide useful anatomical information for treatment planning of surface mould brachytherapy, total body irradiation, and total skin electron therapy, for example, without delivering any imaging dose.

**Methods:**

A 2D pix2pix GAN was trained on 15,000 axial MRI slices of healthy adult brains paired with corresponding external mask slices. The model was validated on a further 5000 previously unseen external mask slices. The predictions were compared with the “ground-truth” MRI slices using the multi-scale structural similarity index (MSSI) metric. A certified neuro-radiologist was subsequently consulted to provide an independent review of the model’s performance in terms of anatomical accuracy and consistency. The network was then applied to a 3D photogrammetry scan of a test subject to demonstrate the feasibility of this novel technique.

**Results:**

The trained pix2pix network predicted MRI slices with a mean MSSI of 0.831 ± 0.057 for the 5000 validation images indicating that it is possible to estimate a significant proportion of a patient’s gross cranial anatomy from a patient’s exterior contour. When independently reviewed by a certified neuro-radiologist, the model’s performance was described as “quite amazing, but there are limitations in the regions where there is wide variation within the normal population.” When the trained network was applied to a 3D model of a human subject acquired using optical photogrammetry, the network could estimate the corresponding MRI volume for that subject with good qualitative accuracy. However, a ground-truth MRI baseline was not available for quantitative comparison.

**Conclusions:**

A deep learning model was developed, to transform 3D optical scan data of a patient into an estimated MRI volume, potentially increasing the usefulness of optical scanning in radiation therapy planning. This work has demonstrated that much of the human cranial anatomy can be predicted from the external shape of the head and may provide an additional source of valuable imaging data. Further research is required to investigate the feasibility of this approach for use in a clinical setting and further improve the model’s accuracy.

## Introduction

Three-dimensional (3D) optical and infrared surface scanning in radiation oncology is increasingly being investigated for determining the 3D exterior surface of a patient without the need for a computed tomography (CT) scan. This technology is often utilised for surface-guided radiation therapy (SGRT) to assist with patient positioning and real-time motion management [1–3]. While this approach most often utilises structured light scanning (SLS) to determine the patient’s external contour, other types of optical surface scanning such as photogrammetry or Lidar (light detection and ranging) have been investigated for applications in radiation oncology [4].

Interest in these various technologies has generally been for the design of 3D printed customised medical devices such as bolus and surface mould applicators for brachytherapy [5–10]. Research into 3D printed beam modifying devices using optical surface scanning has increased in recent years, with several studies demonstrating the feasibility of photogrammetry and SLS for producing brachytherapy surface applicators with similar dosimetric properties to conventional CT derived applicators [8, 9, 11].

In 2019, Douglass and Santos demonstrated that photogrammetry could be used to define the surface of a patient using photogrammetry and 3D print a superficial bolus [12]. The same year, LeCompte et al. [10] used an Apple® iPhone X to produce a 3D model and superficial bolus of a nose. In 2021, Bridger et al. investigated the effect of camera type and settings on the reconstruction accuracy of photogrammetry for radiation therapy applications [13]. A paper published in 2022 by the same group demonstrated that the dosimetry of a 3D printed superficial brachytherapy applicator achievable using photogrammetry was almost equivalent to that of a conventional CT-derived applicator [8].

Unlike conventional radiographic imaging modalities like CT and MRI, optical surface scanning can provide textural colour information about a patient, potentially enabling PTV surface delineation for superficial treatments without marker wires. Photogrammetry and Lidar [4] are cost-effective methods of generating 3D models of a patient’s anatomy and are now available on some consumer smartphones.

Maxwell et al. investigated various scanning technologies, including CT, photogrammetry, and 3D scanners, and found surface scanning technologies superior to CT and photogrammetry [9]. However, in a recent editorial, it was shown that photogrammetry might produce exterior patient contours of higher spatial accuracy than that of CT [4]_._

Crowe et al. proposed a method of generating synthetic homogeneous water equivalent CT datasets for planning radiotherapy treatments using SLS alone, which may be helpful for some treatment techniques such as TSET or TBI but is still limited because of a lack of internal anatomical information [11].

These recent works have demonstrated that optical surface scanning may be helpful in combination with other imaging modalities, or alone in a superficial treatment workflow. However, the lack of internal anatomical information provided by optical surface scanning (OSS) technologies limits its adoption for all but a limited number of radiotherapy techniques and sites.

The current study aimed to investigate whether OSS, combined with deep-learning (DL) techniques, could provide additional anatomical information, increasing the usefulness of OSS for other radiation therapy modalities and for additional sites. Deep learning is useful for automating various tasks in radiation oncology, most notably organ segmentation but has also been used for dose calculations and linear accelerator quality assurance [14–21]. Generative deep learning models such as generative adversarial networks have been used in other domains to perform image-to-image translation tasks such as: converting photographs taken during the day to night, converting hand-drawn pictures into realistic photos, and converting satellite images into maps [22–27]. They have also been used in medical applications to perform tasks such as converting MRI datasets into synthetic CT datasets [28–34].

In the current work, we investigate whether optical surface scanning technologies such as photogrammetry and Lidar from a typical smartphone combined with deep learning models could be used to estimate the probable anatomy of a patient from only their exterior contour. The deep learning model was designed to produce a synthetic MRI (sMRI) for a given patient, which could be used for planning some treatment sites or allow a preliminary plan to be optimised before the primary CT dataset is available.

The objectives of this project were to train a deep learning model to convert the external contour of a patient from photogrammetry and generate an estimated sMRI dataset. This model would then be validated to ensure the model’s predictions agreed with the validation dataset. The validated model would then be tested on an actual 3D optical scan of a patient to demonstrate the model’s usefulness.

## Method

### Data Preparation

581 T1 weighted MRI images of healthy adult subjects were used from the Information eXtraction from Images (IXI) database [35] comprising data obtained from three hospitals.

The MRI volumes, available in NIFTI format, were first resampled in Slicer [36] into a uniform voxel resolution of 1 mm × 1 mm × 1 mm from the original resolution of 1 mm × 1 mm × 1.2 mm. The resampled volumes were then imported into MATLAB 2020b (The MathWorks, Inc., MA, USA).

For each MRI image slice, the *imadjust* function in MATLAB was applied, which, by default, saturates the bottom 1% and the top 1% of all pixel values. This operation increases the contrast of the original image. A threshold operation was then applied to the image such that all pixels with an intensity greater than 20 out of a maximum of 256 (8-bit image) were considered part of the external mask. MATLAB’s *imfill* operation was then applied to fill holes in the binary mask for each slice. A morphological erosion operation was then applied to the mask using MATLAB’s *imerode* function with a radius of 3 and decomposition of 0. These settings were obtained through an iterative process until a suitable mask of the patient’s external contour was obtained for each image slice.

**Fig. 1:**
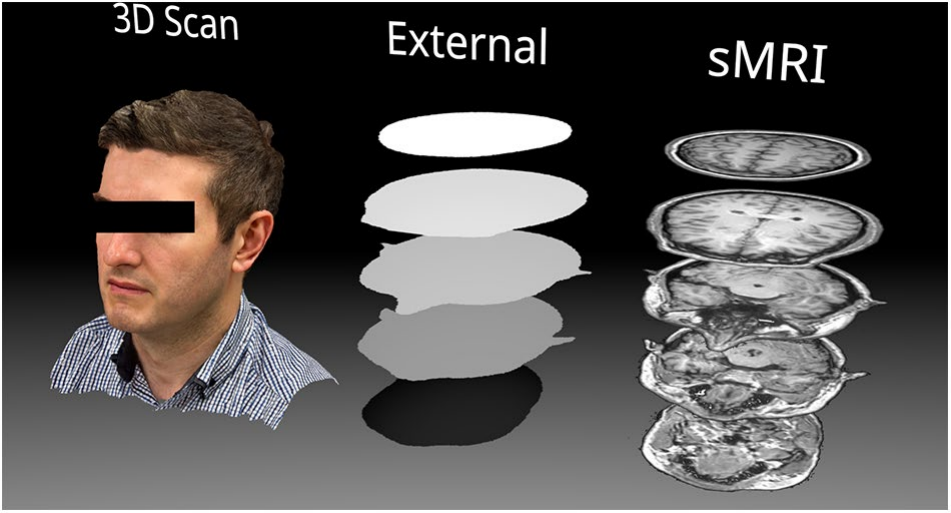
Proposed workflow. Left: The 3D scan of the patient acquired using optical scanning is converted into an external mask (middle) with grayscale values to indicate the relative slice position in the superior-inferior axis. This data is then used as an input to the GAN model to predict the internal cranial anatomy’s synthetic MRI (sMRI)

In the current work, a 2D deep learning model was implemented, which lacked spatial awareness of neighbouring axial slices. To overcome this limitation, the external mask slices were encoded with relative slice position information by adjusting the pixel intensity value of the binary mask slices with values from 1 to 256, indicating the relative position of the slice (Fig. 1) in each MRI volume in the axial direction (inferior most slice binary pixel intensity was one and superior most slice had a binary value of 256). This approach enabled the 2D deep learning model to “know” approximately where the external contour slice was in the patient’s head without using a 3D deep learning model.

The MRI and mask volumes were separated into individual axial slices producing a total of 15,000 slices for the training set and a further 5,000 slices for the validation set. Each patient’s MRI volume was sliced into 256 axial slices for training and testing, equating to approximately 60 unique patients in the training set. The MRI and external mask slices were resized to 256 × 256 pixels to match the input and output resolution of the deep learning model. The mask and MRI slices were then converted to single channel 8-bit greyscale images to train the model.

### Deep learning model training

A 2D pix2pix generative adversarial network (GAN) deep learning model was implemented in MATLAB version 2020b based on the work of Isola et al., [37] by adapting the code from MATLAB’s deep learning GitHub repository [38].

This deep learning model uses a conditional generative adversarial network to convert an image from one domain to another by learning the transformations between a set of paired image data, in our case, external masks of a patient and their corresponding MRI slices. The model developed in the current work takes axial external mask slices of patients with a resolution of 256 × 256 as an input and produces a corresponding, axial MRI slice of the same dimensions.

The deep learning model was trained for 20 epochs on an Nvidia® (Santa Clara, CA) Quadro RTX 4000 GPU. The training of the generator model was evaluated in terms of a combined mean absolute error and cross-entropy loss functions using the Adam optimiser as described in the original pix2pix GAN paper [37, 38].

### Model validation

The model was validated using 5000 ground-truth MRI and binary mask slices, which the model had not previously seen. The predictions of the trained model were compared with the ground-truth MRI scans. The ground-truth and predicted MRI slices were compared visually using the *imfuse* function in MATLAB, which highlights differences in image intensities as two separate colour channels.

To quantify the similarity between the predicted and ground-truth MRI slices, the *Multi-Scale Structural Similarity Index (MSSI)* in MATLAB was used for each pair of predicted and ground-truth slices in the validation set. A value of one indicates perfect structural similarity and is only possible if the test and reference images are identical. This metric was chosen because, unlike other metrics such as mean squared error (MS), which only considers differences in pixel intensities, the MSSI compares structural similarities and has been shown to correlate well with human perceived similarities and is therefore well suited for comparing two MRI scans [39].

A certified neuro-radiologist was also consulted to independently review a subset of the predicted MRI images from the validation set and comment on the anatomical accuracy and consistency of the predictions. The neuro-radiologist had no prior knowledge or input in developing or testing the deep learning model and, therefore, could provide an objective critique of the model’s performance.

### Evaluation of Model on Photogrammetry Data

To demonstrate this proposed workflow on a human subject, a photogrammetry scan of one of the authors was taken using an iPhone 13 Pro and the Metascan© photogrammetry app. The photogrammetry scan was produced by taking 192 photos from approximately equal equatorial angles and three azimuthal angles. The 3D scan was reconstructed in high-quality mode and exported from Metascan© in OBJ format (a standard 3D model format). The model containing 695 k triangular faces was imported into the 3D modelling tool Blender [40]. Since the GAN model was trained on MRI data which contained no information about the patient’s hair, the hair from the photogrammetry scan needed to be artificially removed to ensure the model did not interpret the hair volume as part of the external mask. The sculpting tools in Blender were used to estimate and correct the geometry of the subject’s scalp to approximate a shaved head.

The modified 3D model was then imported into Slicer to generate a voxelised external mask from the 3D model. The mask was resampled to a uniform voxel size of 1 mm × 1 mm × 1 mm and then cropped to slices of resolution 256 × 256 pixels. The masked slices were then sliced axially, and the binary labels for the external mask were encoded with greyscale information to represent the relative axial position of each slice in the head. The mask slices were then evaluated by the trained GAN model one at a time to produce a sequence of predicted MRI slices.

Since no reference MRI scan was available for the author used as the test subject, a quantitative or qualitative comparison was not possible.

## Results

### Model training and validation

**Fig. 2:**
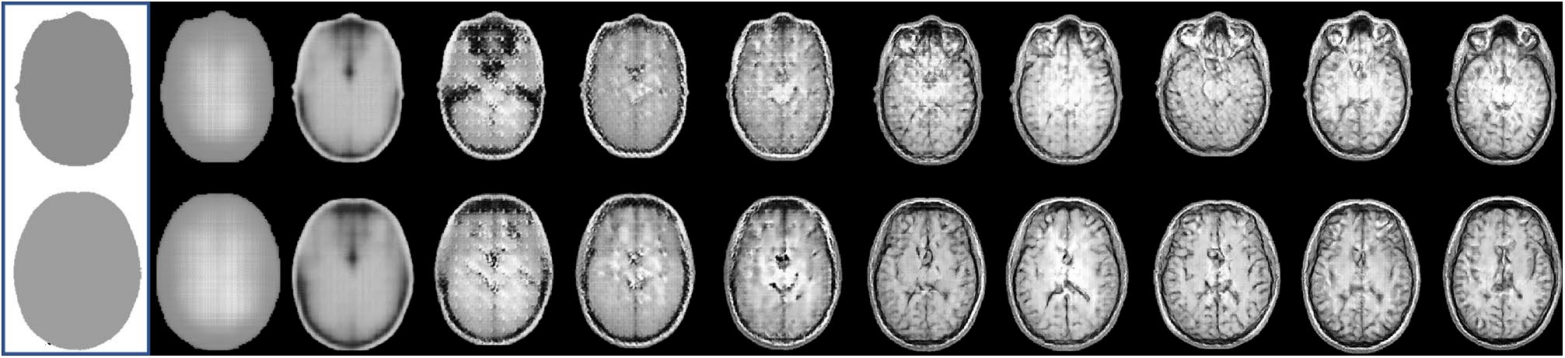
Example of the training process performed in MATLAB 2020b. The images on the left shows the external masks for two example axial slices. The subsequent images on each row show the prediction for each external mask after several iterations of training. The right most images show the predictions after two epochs

**Fig. 3:**
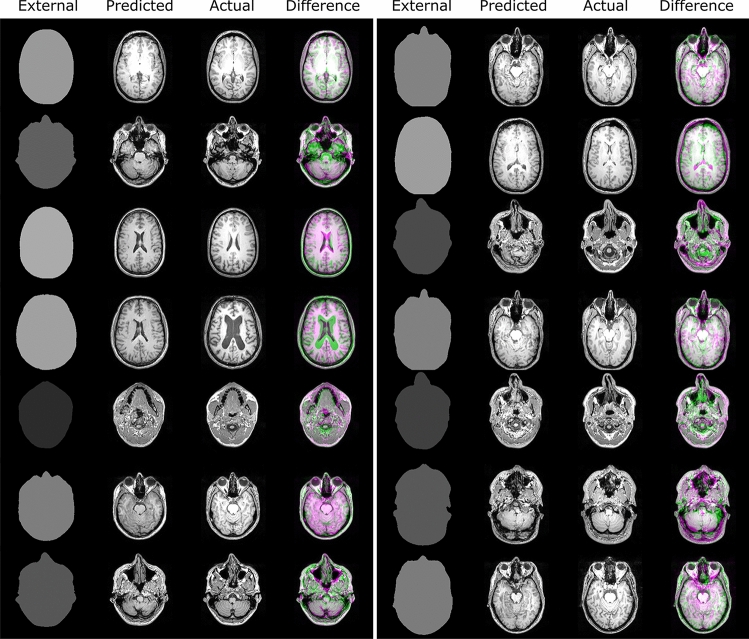
Examples of the validation results showing the performance of the pix2pix GAN model compared with the ground-truth MRI data. A greyscale region in the difference figure highlights regions of similarity between the two images. In contrast, a pink or green colour indicates higher pixel intensity in the ground truth and predicted MRI slices, respectively

The deep learning model was trained for 20 epochs requiring approximately 48 h on an Nvidia® Quadro RTX 4000 GPU. The relative improvement in the model's predictions with time during training is shown in Fig. 2. A visual comparison of a subset of the validation images is shown in Fig. 3 and shows a high degree of visual similarity in terms of the anatomical structure between the ground-truth and predicted MRI slices. In many cases, much of the perceived difference between the images is due to a relative difference in pixel intensity rather than a structural difference.

Each of the 5000 predicted MRI and ground truth MRI pairs were compared using the MSSI. The mean, median, and standard deviation of the MSSI scores were calculated and displayed as a histogram. MRI slices corresponding to regions outside the external contour of the patient were excluded from the analysis leaving 4255 image pairs. This was due to the high number of images with an MSSI of approximately 1.0. These images correspond to each patient’s superior and inferior slices, which contain no anatomical structure and are structurally very similar.

**Fig. 4:**
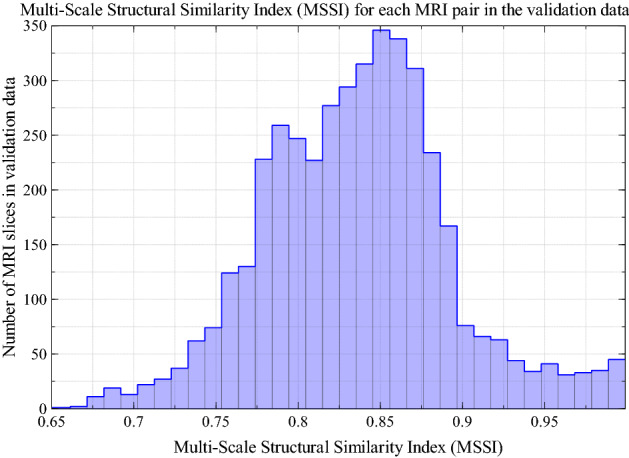
A comparison of structural similarity between the ground-truth MRI slices and those predicted by the deep learning model. A value of 1 indicates that images are identical. Slices outside the region belonging to the external mask have been excluded from the plot (i.e., empty slices)

The mean and standard deviation for the MSSI was 0.831 and 0.057, respectively, and the median value was 0.832. A histogram showing MSSI values for all validation images, excluding those outside the patient volumes, can be seen in Fig. 4.

A certified neuro-radiologist with no prior contribution to the development of the model reviewed a subset of the predicted MRI images and compared them with the ground-truth images to check for anatomical accuracy and consistency. The following observations were made about the model’s performance and accuracy of the predictions.

The cranial vault is quite accurately predicted postero-superiorly. There is a limitation in predicting the cranial vault anteriorly and antero-inferiorly in the frontal sinus region. There is significant variation in the size of pneumatizedtized frontal sinus in the normal population. Hyperostosis of the inner table (increased thickness) can occur within the population and is more common in females. The posterior skull base is difficult to accurately predict due to the considerable variation in the size of the temporal bones and shape of the lower occipital bone (which is related to cerebellar size). The misregistration is most pronounced inferiorly at the level of the foramen magnum. The central skull base is moderately well done, except that prediction of the sphenoid sinus pneumatizationation and shape of the sphenoid wings is difficult due to considerable variation in the population. The anterior skull base is again difficult due to variability in the size (including width) of the ethmoid sinuses, and the size of the lower frontal sinuses/orbital roof position. The orbital contents, including the globes, are quite well predicted. There will be some variation in the size and shape of the orbits between races. Cortical brain position in relation to the inner skull table is reasonably well done except for anterior frontal and sub-frontal regions as discussed above (dependent on frontal sinuses etc.) There will be some variation in cortex position relative to the inner table with age due to expected brain volume loss. The brainstem is reasonably well done. Cerebellar size and shape are difficult (vermis to cisterna magna relationship) as they vary within the population. Lateral ventricular size is difficult to predict due to large variations within the population. Third ventricular size also varies. The fourth ventricle is predicted reasonably well. It would be difficult to predict the gyral folding pattern of the brain due to the large variation within the normal population.

Outer head-to-brain angulation is quite good. Spatial resolution is quite good. Images are not too noisy, allowing for some expected misregistration—no significant artefacts.

In summary, the machine learning model predicts the regions that exhibit mild variation in humans quite well. It would be impossible to accurately predict regions that exhibit significant variation in humans, such as the size of the paranasal sinuses, temporal bones, and lateral ventricles, from just an external contour.

### Model evaluation on photogrammetry data

**Fig. 5:**
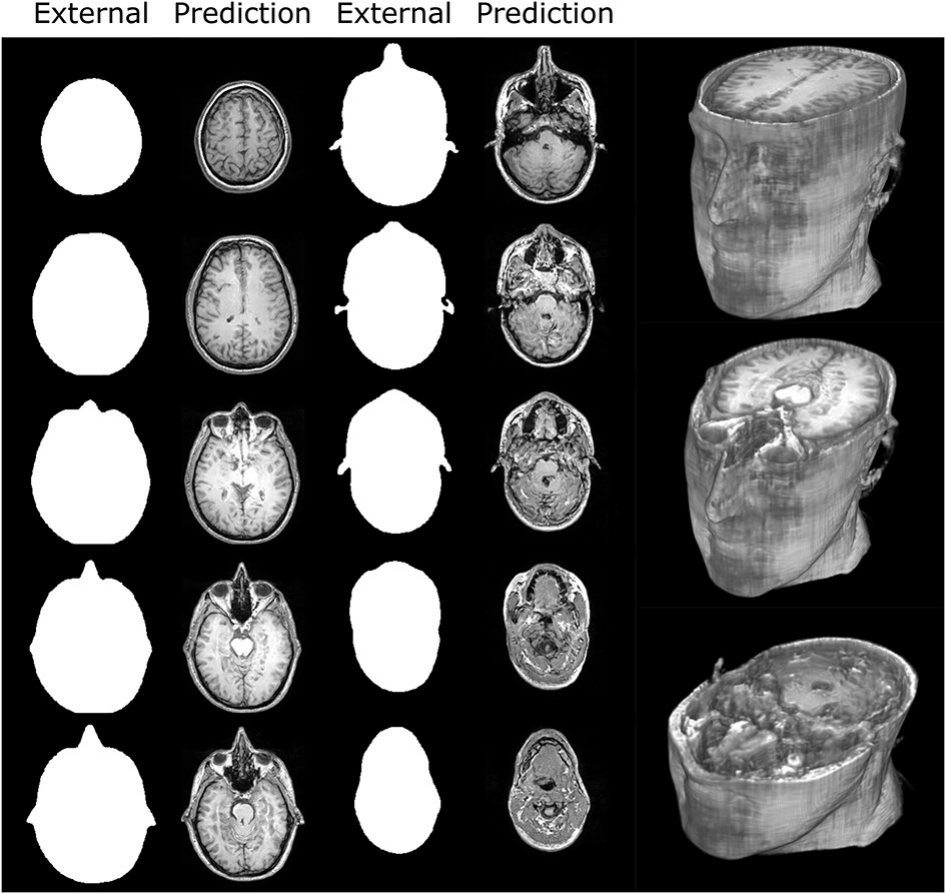
Examples of the external mask slices and corresponding predicted MRI data obtained using photogrammetry of a human subject. Some volumetric renders are shown on the right with cutaways at three different axial levels

A photogrammetry model of the author was processed slice-by-slice in the axial direction through the pix2pix GAN to produce MRI predictions for each slice. Some examples of the masks and corresponding MRI predictions as well as some volumetric data are shown in Fig. 5. Since a reference MRI dataset was not available for comparison, a quantitative analysis of the predictions was not possible, however, visually, the predicted MRI volume showed a surprisingly high degree of continuity between axial slices despite being generated using a 2D model. Major anatomical features of the brain were present in the correct locations and appeared convincing to an untrained observer.

## Discussion

A deep learning model was trained in the current work to transform 3D optical scan data of a patient into an estimated MRI volume, potentially increasing the usefulness of optical scanning and SGRT in radiation therapy. While this preliminary model produces predictions of good quantitative accuracy, the approach is not without its limitations.

The model was trained on a healthy adult brain MRI dataset and will not generalise to other sites without additional data and training. This dataset was trained on an open-source dataset of healthy adults, which is generally difficult to find for other sites and would require a more extensive study to acquire the necessary data. Data of healthy patients is limited, and data containing tumours and other diseases results in the GAN network trying to generate these volumes in the synthetic image slices. This could, in theory, be overcome by manually removing slices containing tumours from the training data to ensure the model does not learn these features. However, since the model cannot localise tumours from a patient’s external volume, the model’s usefulness may be limited to a few select treatment techniques and sites without additional complimentary imaging data for each patient.

The MRI-style output of the model limits its application in radiation therapy treatment planning due to the lack of electron density information necessary for dose calculations. Ideally, future models should be trained on CT data to expand the usefulness of such a model.

Using a 2D pix2pix GAN resulted in diminished continuity of anatomical features between predicted MRI slices because of the limited spatial awareness of neighbouring slices provided by the greyscale encoding system. A 3D model implementation would likely improve slice continuity and provide better spatial awareness for the model. However, a 3D model would likely take longer to train and require additional training data to achieve this improved performance.

Exterior contours obtained from Lidar or photogrammetry measurements would, in most cases, contain facial hair compared to a CT measurement. As a result, the GAN model attempts to fill the slices containing hair with normal brain tissue. In the current work, the 2D model was trained so that the model would not use information about the slices containing hair and influencing neighbouring slices. The axial slices containing the hair could simply be excluded as unreliable predictions. It is unclear without further investigation whether a 3D implementation of this model would account for the hair volume more robustly. Dedicated training data consisting of photogrammetry-derived external contours and matched/aligned MRI/CT volumes would likely partially overcome this issue.

The external mask generated from the MRI training data to define the patient’s skin was based on an arbitrary pixel intensity value and subject to uncertainty. As a result, the external mask from MRI may differ from that generated using Lidar or photogrammetry, resulting in uncertainties in the predicted output. While the validation results indicate this model performs accurately and has the potential to provide practical estimates of the internal anatomy of patients, further work is required to determine if the same model can be used to estimate cranial anatomy from photogrammetry, Lidar, or structured light scanning without additional curated data. Ideally, a more extensive study involving the collection of MRI and matched photogrammetry data would be required to verify the model’s accuracy for this intended use case.

The model was trained on 8-bit image data to reduce training time, limiting the maximum grey scale values in the predicted output to 256. Future models could be trained on 12 or 16-bit MRI or CT data to be more consistent with conventional DICOM images and enable this image data to be used in a treatment planning workflow.

### Suggested use cases

The rationale for the current work was to expand upon our previous works [4, 8, 12, 13] and the work of S. Crowe et al. [9, 11], who suggested a homogeneous water equivalent CT dataset from a structured light scanner for planning some radiotherapy treatments. The apparent limitation of this approach is that it produces no anatomical information for planning, limiting its usefulness. The approach demonstrated in the current work increases the usefulness of photogrammetry and other optical scanning techniques in radiation therapy as it provides an estimate of this anatomy.

Some proposed use cases for this technique include preliminary planning of radiation therapy treatments before the primary planning CT is available and re-optimised once available. This may be particularly useful for patients living in rural areas with long commute times to the treatment centre. This approach may also be helpful for adaptive planning workflows. Many centres are already using SGRT technologies that could be combined with this model to predict anatomical changes after weight loss or patient movement. This approach could also enable estimates of a patient’s internal anatomy for real-time motion management.

Some centres are currently investigating upright seated patient treatments for proton therapy using fixed beamlines. Cone-beam CT imaging in this position is technically and logistically complex; the approach suggested in the current work could be a valuable part of the solution to such a problem.

Sites where this technique could conceivably be used for treatment planning without additional imaging data include total body irradiation, total skin electron therapy, surface mould brachytherapy, and whole brain or palliative radiotherapy for non-malignant disease.

In addition to the clinical applications, this approach could enable realistic synthetic imaging datasets to be created for research or training by simply manipulating the external contour of the geometry.

### Suggestions for future work

Based on our experiences in developing the model of the current work, we would suggest the following approaches be applied when developing future models of this type.

The brain is a complex anatomical site with significant variation amongst the general population. This site was not ideal for this preliminary model, and this site was only chosen because of the availability of training data consisting of healthy patients without disease. This model may perform more accurately for sites such as thorax or extremities where there is reduced inter-patient variability in the underlying anatomy.

Most importantly, to produce a model of sufficient accuracy to be considered clinically useful, a more extensive clinical study involving the collection of optically scanned 3D models of patients and their corresponding registered MRI or CT data to translate directly between these imaging domains is required.

Since optical scanning techniques generally include textural information in addition to the geometric data, an investigation could also be performed to see if this information can be used to augment the model’s performance.

As more accurate models are developed, an investigation should be performed to compare relevant organs at risk contours between the sMRI and MRI ground truths to see if the changes in predicted contours are clinically significant. These differences could be assessed using metrics such as the dice coefficient, for example. A side-by-side comparison of existing treatment planning techniques for treatments such as TSET, TBI, and surface mould brachytherapy with this proposed technique would also provide valuable insight.

## Conclusion

It has been demonstrated that optical surface scans of the exterior contour of a patient can, in principle, be used to estimate the interior anatomy of a patient with some degree of confidence. The mean multi-scale structural similarity index on the validation dataset was approximately 0.83 indicating a good agreement between the ground-truth MRI images and predictions by the pix2pix GAN network.

While further research is required to improve the model's performance to a point where it could be clinically useful, this study has demonstrated that, in principle, this approach is feasible. Various optical surface scanning technologies are already used clinically for SGRT techniques which could be utilised in our proposed workflow. Low-cost smartphone-based optical scanning technologies such as photogrammetry and Lidar enable further possibilities for this approach.
